# Factors influencing nursing students’ knowledge, attitudes, and infection management behavior for emerging respiratory infectious diseases: A cross-sectional study

**DOI:** 10.1371/journal.pone.0290874

**Published:** 2023-08-31

**Authors:** Ha-Young Park, In-Sun Yeom

**Affiliations:** 1 College of Nursing, Kyungbok University, Namyangju-si, Gyeonggi-do, Republic of Korea; 2 College of Nursing, Sungshin Women’s University, Gangbuk-gu, Seoul, Republic of Korea; University of Hafr Al-Batin, SAUDI ARABIA

## Abstract

Understanding the factors influencing infection management behavior related to Emerging Respiratory Infectious Diseases (ERIDs) among nursing students is important because nursing students play a vital role in preventing the spread of ERIDs. This study aimed to determine factors influencing infection management behavior related to ERIDs among nursing students in Korea. In this cross-sectional survey study, data were collected from May 3 to June 30, 2022, using a questionnaire. Data collected from 481 nursing students were analyzed using descriptive statistics, an independent t-test, a one-way analysis of variance, Pearson correlation coefficients, and hierarchical multiple regression using the SPSS program. The key factors influencing respondents’ ERID-related infection management behavior were attitude (β = 0.554, *p*<0.001) and knowledge (β = 0.282, *p*<0.001). The explanatory power of this model was 40.2%, and the regression model was found to be statistically significant (F = 27.935, *p*<0.001). To improve nursing students’ ERID-related infection management behavior, their knowledge of and attitudes toward ERIDs should be enhanced by repeatedly providing them with accurate professional information about ERIDs. Therefore, intensive efforts should be taken to develop a systematic educational program that can help nursing students better perform infection management.

## Introduction

Emerging Respiratory Infectious Diseases (ERIDs) are caused by infectious agents that cause acute respiratory complications [1]. Some representative examples of ERIDs are Severe Acute Respiratory Syndrome (SARS), Middle East Respiratory Syndrome (MERS), and Coronavirus Disease 2019 (COVID-19). The emergence of these ERIDs poses a serious threat to people’s lives and to economies and societies at a global level [1]. South Korea experienced a MERS outbreak in 2015, and as of December 30, 2022, the total cumulative confirmed cases of COVID-19 amounted to 28,996,3477, with a death toll of 32,095 (fatality rate of 0.12%) [2]. Currently, South Korea is still not free from ERIDs. Under these circumstances, efforts related to the prevention and control of ERIDs are gaining increasing importance worldwide, and particular attention is paid to the role of the nursing workforce, which plays the most important role during an ERID pandemic [3–5].

Nurses are a key workforce performing infection control tasks in healthcare facilities to protect patients, caregivers, employees, and visitors from infection, in addition to providing direct nursing care to infected persons, with limited resources in ERID-caused disaster situations [3–5].

However, the biggest problem during an ERID pandemic is that most nurses are often assigned frontline work in the event of ERID outbreaks without being properly trained with professional knowledge and skills, including related to quarantine procedures and methods, appropriate use (wearing and removal) of Personal Protective Equipment (PPE), and practical nursing guidelines for patients with infectious diseases [6, 7]. Consequently, nurses experience not only physical stress due to the excessive work involved in caring for patients with ERIDs, which they may not be accustomed to, but also mental stress due to extreme anxiety about the risk of infection [7].

For more effective management and response to an ERID-caused pandemic, it is important to improve nurses’ infection management behavior. To this end, great importance is attached to the need for systematic and repetitive ERID education in university nursing curricula [8, 9]. Therefore, efforts will have to be redoubled to continuously improve ERID-related infection management behavior among clinical nurses as well as nursing students, who constitute the future nursing workforce.

Previous research has demonstrated that clinical nurses’ ability to perform infection control against ERIDs increases with their knowledge level and positive attitude related to managing ERIDs [10]. However, in South Korea, no research has yet been conducted to identify the factors associated with nursing students’ ERID-related infection control performance, although recent studies have determined the relationship between knowledge, attitudes, and risk perceptions among healthcare students regarding COVID-19 prevention behavior [11].

In an attempt to bridge this research gap, the current study aimed to determine nursing students’ ERID-related knowledge, attitudes, and infection management behavior in preparation for a possible resurgence of ERIDs and identify the factors influencing their infection management behavior with a view to preparing source data for the development of ERID infection control education programs. Specifically, the following objectives were pursued: First, we determined participants’ ERID-related knowledge, attitudes, and infection management behaviors. Second, we determined the inter-participant differences in ERID-related knowledge, attitudes, and infection management behavior depending on participant characteristics. Third, we examined the correlations between the participants’ ERID-related knowledge, attitudes, and infection management behavior. Finally, the factors influencing participants’ infection management behavior were identified.

## Methods

### Study design

This cross-sectional survey was conducted to determine the factors influencing nursing students’ ERID-related knowledge, attitudes, and infection management behavior.

### Ethics statement

This study was approved by the Korean Public Institutional Review Board of the Ministry of Health and Welfare (No. P01-202205-01-012). The study was conducted after receiving approval and the data were collected from May 3 to June 30, 2022.

### Participants

Participants were recruited through convenience sampling from among first- to fourth-year nursing students enrolled in the Department of Nursing in Kyungbok University, Korea. Participants were recruited from March to May 2022. However, due to the COVID-19 pandemic in Korea, all universities were conducting online classes, and social distancing made it difficult to properly explain our study and recruit participants for our study. Therefore, we used convenience sampling to recruit participants.

The participants were provided information on the purpose of the study and they agreed to participate voluntarily. The sample size appropriate for the multiple regression analysis in this study was 387, when calculated using the G*Power 3.1.9 program, with the significance level set at 0.05, power at 95%, effect size at 0.06, and the number of predictor variables at eight (two independent variables: knowledge and attitude; five participant characteristics: age, grade, living with family, clinical practice, and PPE-related training). The effect size was set at 0.06 considering the lack of previous ERID-related research. Additionally, to compensate for a dropout rate of approximately 20% [9, 10], a total of 484 questionnaires were distributed (121 per grade level), of which 481 were used for analysis after removing three questionnaires with incomplete responses.

### Research instruments

The questionnaire consisted of 66 items (19 general items, 20 items on ERID-related knowledge, 12 items on attitudes toward ERIDs, and 15 items on infection management behavior toward ERIDs), and it took approximately 15 min to complete.

#### Knowledge about ERIDs

To measure ERID-related knowledge, a 20-item scale developed by Choi and Lee [10] was used after adapting it to suit the current study’s purpose. To establish the content validity of the revised scale, Item-Level Content Validity Index (I-CVI) testing was conducted by a five-member expert panel comprising one nursing professor, one infectious disease physician, and three infection control nurse specialists. The I-CVI scores obtained were 0.8 or higher for all 20 items grouped into four categories: cause and latency (four items), propagation route and diagnostic criteria (four items), symptoms and treatment (four items), and imposing and lifting quarantine (eight items). Each item is rated on a binary scale (incorrect answer = 0, correct answer = 1), with a total score ranging from 0 to 20 points, where a higher total score indicates a higher level of knowledge. The Kuder-Richardson Formula 20 (KR 20) was 0.68 in Choi and Lee’s study [10] and 0.61 in the current study [12].

#### Attitudes toward ERIDs

To measure attitudes toward ERIDs, a 12-item scale developed by Choi and Lee [10] was used after adapting it to suit the purpose of the present study. To establish the content validity of the revised scale, I-CVI testing was conducted by the same expert panel mentioned above. The I-CVI scores obtained were 0.8 or higher for all 12 items. Each item is rated on a 5-point Likert scale (1 = strongly disagree, 5 = strongly agree), where a higher total score indicates a more positive attitude toward ERIDs. The instrument reliability (Cronbach’s α) was 0.83 in Choi and Lee’s study [10] and 0.72 in the current study [13].

#### Infection management behavior toward ERIDs

To measure infection management behavior toward ERIDs, a 15-item scale developed by Choi and Lee [10] was used after adapting it to suit the purpose of this study. To establish the content validity of the revised scale, I-CVI testing was conducted by the same expert panel mentioned above. The I-CVI scores thus obtained were 0.8 or higher for all 15 items. Each item is rated on a 5-point Likert scale (1 = strongly disagree, 5 = strongly agree), with the total score obtained by adding up the item scores; a higher total score indicates a higher level of infection management behavior toward ERIDs. The instrument reliability (Cronbach’s α) was 0.95 in Choi and Lee’s study [10] and 0.97 in the current study [13].

### Data collection

A recruitment announcement was posted on the online bulletin board of the Department of Nursing in Kyungbok University, and nursing students wishing to participate in the study were instructed to participate by email. Those who agreed to participate in the questionnaire survey were provided written information (by email) on the purpose and methods of the study and compensation for participation. A link to the Google online survey form was then sent to those who provided written informed consent to participate in the study. To protect the rights of the participants, the anonymity of the collected data and exclusive use of data for research purposes were guaranteed, and explanations were given regarding participants’ right to withdraw from the study at any time without incurring any disadvantages.

### Data analysis

The collected data were analyzed using the SPSS/Win (Ver. 27.0, SPSS Inc., Chicago, IL, USA) program. Participants’ general characteristics were analyzed as real numbers, frequencies, and percentages. Knowledge, attitudes, and infection management behavior toward ERIDs were analyzed in terms of means and standard deviations. The ERID-related knowledge, attitudes, and infection management behavior depending on participant characteristics were analyzed using an independent t-test and one-way analysis of variance (ANOVA), and post-hoc analysis was performed using the Bonferroni post-hoc test. Correlations between the participants’ ERID-related knowledge, attitudes, and infection management behavior were analyzed using Pearson’s correlation coefficient. Factors influencing participants’ infection-management behavior were analyzed using hierarchical multiple regression analysis. The scores of knowledge, attitudes, and infection management behavior toward ERIDs were standardized before the hierarchical multiple regression analysis.

## Results

### Participant characteristics

The 20s was the predominant age group of the participants (85.7%). The number of participants classified by school year was 118 freshmen (24.4%) and 121 sophomore, juniors, and seniors each (25.2%). Female students outnumbered male students (88.6 vs. 11.4%). Students with no religion outnumbered students with a religion (52.6% vs. 47.4%). Most of the students were living with their families (94.8%), 56.5% had experience of clinical practice, 43.7% had experience of exposure to ERIDs, 74.4% had received ERID-related education, and 73.8% had received PPE-related training.

### Knowledge about ERIDs

The mean ERID knowledge score was 18.29±1.29 out of 20. The correct answer rate was 91.46%; the items that obtained the highest correct answer rate (99.2%) were “ERIDs include diseases such as MERS and COVID-19” in the category “cause and latency” and “Hand hygiene should be performed immediately after removing PPE” and “When disinfecting the space used by a confirmed ERID patient, a health mask (KF 94) and PPE such as gloves should be worn before starting disinfection, and touching the eyes, nose, and mouth should be avoided while disinfecting” in the category “imposing and lifting quarantine.” The item that obtained the lowest correct answer rate (49.3%) was “The criteria for lifting quarantine for confirmed COVID-19 cases vary depending on the patient’s symptoms, whereby the absence of clinical symptoms is usually judged as no risk of contagion,” in the category “imposing and lifting quarantine” (Table 1).

**Table 1:** Knowledge about ERIDs (N = 481).

Category	True/False	Correct (%)	M±SD
**Cause and latency**			
ERID^a^ is an infectious disease with an endemic or imported source of infection.	T	96.3	3.90±0.35
The main cause of ERID is viruses.	T	98.1	
ERIDs include diseases such as MERS^b^ and COVID-19^c^.	T	99.2	
The incubation period of COVID-19 is 1–14 days (5–7 days on average).	T	96.9	
**Propagation route and diagnostic criteria**			
ERIDs can spread through various routes such as droplets, air, and contact.	T	93.1	3.70±0.52
ERIDs can be easily transmitted in closed, crowded, or poorly ventilated settings.	T	78.8	
Hand washing with water and soap for at least 30 s can help prevent the spread of ERIDs.	T	99.6	
COVID-19 infection is confirmed when found positive in the nasopharyngeal swab PCR^d^ test (gene amplification), in which a sample is collected by inserting a cotton swab into the nose.	T	98.8	
**Symptoms and treatment**			
In the early stages of COVID-19 infection, mild respiratory symptoms (e.g., cough, fever, and sore throat) often appear.	T	99.0	3.39±0.64
COVID-19 infection does not necessarily lead to serious symptoms such as pneumonia or shortness of breath.	T	87.1	
All ERIDs can be treated prophylactically with vaccines.	F	58.2	
In principle, inpatient treatment for early-stage ERID patients should be performed in a hospital equipped with isolated beds or negative pressure rooms.	T	94.8	
**Imposing and lifting quarantine**			
The criteria for lifting a quarantine for confirmed COVID-19 cases vary depending on the patient’s symptoms, whereby the absence of clinical symptoms is usually judged as no risk of contagion.	F	49.3	7.29±0.78
Donning PPE^e^ such as N95 masks, gloves, goggles, and gowns is necessary for caring for patients with ERID.	T	98.8	
If you wear PPE correctly when touching a patient with ERID, there is no need to wash your hands afterward.	F	86.1	
When removing PPE, care should be taken not to come into contact with the outer surface of the PPE items (gloves, gown, mask, face shield), which should then be discarded in a way that causes minimal contamination.	T	99.0	
Hand hygiene should be performed immediately after removing PPE.	T	99.2	
Even in an emergency, exposure to infection should be minimized by wearing PPE.	T	98.8	
When ERID infection is suspected in a patient, PPE should be worn as far as possible until the patient’s route of infection is confirmed.	T	98.8	
When disinfecting the space used by a confirmed ERID patient, a health mask (KF 94) and PPE such as gloves should be worn before starting disinfection, and touching the eyes, nose, and mouth should be avoided while disinfecting.	T	99.2	
**M±SD**	91.46%		18.29±1.29

^a^ERID, emerging respiratory infectious disease

^b^MERS, middle east respiratory syndrome

^c^COVID-19, coronavirus disease 2019

^d^PCR, polymerase chain reaction

^e^PPE, personal protective equipment

### Attitudes toward ERIDs

The mean score for attitudes toward ERIDs was 4.01±0.44. The item that obtained the highest score was “I have experience of refraining from going out when an ERID broke out” (4.50±0.71), and the item that obtained the lowest score was “Wearing PPE to care for an ERID patient causes stress” (2.41±1.05). The responses to “Wearing PPE to care for an ERID patient causes stress” were scored on a scale where 1 = strongly disagree, 2 = disagree, 3 = agree, 4 = agree, and 5 = strongly agree; a lower score indicates a lower level of stress while wearing PPE when caring for an ERID patient (Table 2).

**Table 2:** Attitudes toward ERIDs (N = 481).

Content	M±SD
I think it necessary to educate people around us on ERID-related preventive measures.	4.17±0.90
I thoroughly practice personal hygiene (hand hygiene, cleaning, laundry, etc.) during the outbreak of an ERID^a^.	4.20±0.82
I have experience of checking whether the county I planned to visit was affected by any ERID.	3.09±1.23
I have experience of refraining from going out when an ERID broke out.	4.50±0.71
I have experience of voluntarily searching for new information on ERIDs.	4.25±0.79
I think it increasingly likely that an ERID will break out.	3.91±0.94
I have experience of explaining precautions and prevention measures to people around me during the outbreak of an ERID.	3.88±0.90
I do not worry about food intake because it is safe if I follow the preventive measures against ERIDs.	3.51±1.00
PPE^b^ worn for nursing an ERID patient can protect the patient’s family from infection.	4.21±0.75
PPE worn for nursing an ERID patient can protect me from infection.	4.40±0.65
Wearing PPE to care for an ERID patient causes stress.	2.41±1.05
I will always work with PPE on when an ERID breaks out.	4.36±0.74
**M±SD**	4.01±0.44

^a^ERID, emerging respiratory infectious disease

^b^PPE, personal protective equipment

### Infection management behavior toward ERIDs

The mean score for ERID-related infection management behavior was 4.67±0.51 The item that obtained the highest score was “I will always practice hand hygiene before and after patient care” (4.76±0.54), and the item that obtained the lowest score was “I will explain to patients that they should visit a hospital immediately if they develop respiratory symptoms, such as fever, cough, or trouble breathing, after visiting an ERID risk area or coming into contact with an infected person” (4.46±0.74) (Table 3).

**Table 3:** Infection management behavior toward ERIDs (N = 481).

Content	M±SD
I will explain to patients that they should visit a hospital immediately if they develop respiratory symptoms, such as fever, cough, or trouble breathing, after visiting an ERID^a^ risk area or coming into contact with an infected person.	4.46±0.74
I will always practice hand hygiene before and after patient care.	4.76±0.54
I will always disinfect medical instruments (e.g., thermometers, blood pressure monitors, and stethoscopes) after use.	4.68±0.62
I will wear PPE before coming into contact with a patient at risk of developing an ERID.	4.75±0.54
I will implement precautions against contact, droplet, and airborne infection for confirmed ERID cases according to the guidelines.	4.73±0.57
I will isolate ERID patients in single rooms or negative pressure rooms according to the guidelines.	4.66±0.61
If ERID infection is suspected, I will discuss preemptive quarantine measures.	4.55±0.65
I will post a quarantine sign on the door of the isolation room according to the guidelines; for example, “Caution against airborne, droplet, and contact infection.”	4.70±0.59
I will educate patients and caregivers to refrain from visiting outside of visiting hours.	4.68±0.62
I will don PPE^b^ in a separate place, not in the patient’s room, when I need it to care for an ERID patient.	4.65±0.73
If a patient should be moved, I will assist the patient to wear the prescribed protective equipment, such as a mask.	4.72±0.56
If a patient has to go for a test, I will provide infection information to the department in charge according to the hospital guidelines.	4.68±0.59
I will educate patients to cover their mouth and nose with a tissue when they cough or sneeze, and to practice hand hygiene afterward.	4.69±0.62
I will educate patients and caregivers about the correct measures to ERID infection prevention (practicing hand hygiene, wearing masks, etc.).	4.70±0.57
I will check the latest information on ERIDs.	4.59±0.64
**M±SD**	4.67±0.51

^a^ERID, emerging respiratory infectious disease

^b^PPE, personal protective equipment

### Differences in knowledge, attitudes, and infection management behavior related to ERIDs based on participant characteristics

Significant inter-participant differences in ERID-related knowledge scores were observed depending on grade level (F = 3.190, *p* = 0.023), sex (t = -2.843, *p* = 0.006), and living with family status (t = -2.338, *p* = 0.028). Higher knowledge scores were obtained by sophomores (vs. freshmen), female students (vs. male students), and those living with their family (vs. those living alone).

Significant inter-participant differences in ERID-related attitude scores were observed depending on age (F = 2.671, *p* = 0.047), sex (t = -3.286, *p* = 0.001), living with family status (t = -2.214, *p* = 0.027), and experience of PPE-related training (t = -3.445, *p* = 0.001). Higher attitude scores were obtained by those in their 30s (vs 20s or younger), female students (vs. male students), those living with family (vs. those living alone), and those with experience of PPE-related training (vs. those without).

Significant inter-participant differences in ERID-related infection management behavior scores were observed depending on age (F = 4.605, *p* = 0.003), grade level (F = 6.524, *p*<0.001), sex (t = -2.180, *p* = 0.030), experience of clinical practice (t = -2.011, *p* = 0.045), and experience of PPE-related training (t = -2.493, *p* = 0.014). Higher ERID-related infection management behavior scores were obtained by those aged 20 and over (vs. those younger than 20), second-to-fourth-year students (vs. freshmen), female students (vs. male students), those with experience in clinical practice (vs. those without), and those with experience in PPE-related training (vs. those without) (Table 4).

**Table 4:** Differences in knowledge, attitudes, and infection management behavior related to ERIDs based on participant characteristics (N = 481).

Characteristics	N(%)	Knowledge		Attitude		Infection management behavior	
		M±SD	t or F (*p*)	M±SD	t or F (*p*)	M±SD	t or F (*p*)
**Age**							
<20^a^	14(2.9)	18.07±1.60	0.468 (0.704)	3.66±0.73	**2.671 (0.047) a<c**	4.18±1.10	**4.605 (0.003) a<b,c,d**
20–29^b^	412(85.7)	18.27±1.17		3.90±0.42		4.68±0.47	
30–39^c^	27(5.6)	18.48±1.16		4.06±0.38		4.73±0.36	
>40 ^d^	28(5.8)	18.43±2.47		3.93±0.53		4.68±0.61	
**Grade**							
1^st^ grade^a^	118(24.4)	18.04±1.48	**3.190 (0.023) a<b**	3.83±0.48	2.278 (0.079)	4.50±0.70	**6.524 (<0.001)** **a<b,c,d**
2^nd^ grade^b^	121(25.2)	18.50±1.22		3.95±0.44		4.75±0.45	
3^rd^ grade ^c^	121(25.2)	18.19±1.27		3.96±0.38		4.74±0.38	
4^th^ grade ^d^	121(25.2)	18.29±1.29		3.89±0.43		4.68±0.41	
**Sex**							
Male	55(11.4)	17.64±1.87	**-2.843 (0.006)**	3.73±0.51	**-3.286 (0.001)**	4.53±0.63	**-2.180 (0.030)**
Female	426(88.6)	18.37±1.17		3.93±0.42		4.69±0.49	
**Religion**							
Yes	228(47.4)	18.27±1.36	0.242 (0.809)	3.94±0.42	-1.538 (0.123)	4.66±0.48	0.178 (0.859)
No	253(52.6)	18.30±1.22		3.88±0.45		4.67±0.53	
**Living with family**							
Yes	456(94.8)	18.35±1.15	**-2.338 (0.028)**	3.91±0.42	**-2.214 (0.027)**	4.68±0.48	-1.912 (0.67)
No	25(5.2)	17.16±2.53		3.72±0.60		4.38±0.79	
**Clinical practice experience**							
Yes	272(56.5)	18.35±1.20	-1.287 (0.119)	3.93±0.41	-1.866 (0.063)	4.71±0.39	**-2.011 (0.045)**
No	209(43.5)	18.20±1.39		3.84±0.47		4.61±0.63	
**Exposure to ERIDs** ^e^							
Yes	210(43.7)	18.23±1.35	0.876 (0.381)	3.91±0.49	-0.224 (0.823)	4.61±0.61	1.901 (0.058)
No	271(56.3)	18.33±1.23		3.90±0.39		4.71±0.41	
**ERID-related training**							
Yes	358(74.4)	18.31±1.32	-0.755 (0.450)	3.91±0.45	-0.767 (0.444)	4.67±0.49	-0.741 (0.459)
No	123(25.6)	18.21±1.18		3.88±0.39		4.64±0.57	
**PPE** ^f^ **-related training**							
Yes	355(73.8)	18.30±1.29	-0.415 (0.678)	3.95±0.42	**-3.445 (0.001)**	4.71±0.42	**-2.493 (0.014)**
No	126(26.2)	18.25±1.26		3.79±0.47		4.54±0.69	

^e^ERID, emerging respiratory infectious disease

^f^PPE, personal protective equipment

### Correlations between participants’ knowledge, attitudes, and infection management behavior related to ERIDs

In the correlations between the ERID-related and infection management behavior, knowledge showed a significant positive correlation with attitudes (r = 0.294, *p*<0.001) and infection management behavior (r = 0.319, *p*<0.001). In addition, attitudes showed a significant positive correlation with infection management behavior (r = 0.610, *p*<0.001) (Table 5).

**Table 5:** Relationship between knowledge, attitudes, and infection management behavior related to ERID (N = 481).

	Knowledge	Attitude	Infection management behavior
	r(*p*)	r(*p*)	r(*p*)
Knowledge	1		
Attitude	0.294(<0.001)	1	
Infection management behavior	0.319((<0.001)	0.610(<0.001)	1

### Factors influencing participants’ infection management behavior related to ERIDs

To determine the factors influencing the participants’ infection management behavior, the variables that showed significant inter-participant differences (i.e., age, grade level, sex, living with family, and experience of PPE-related training) were entered into Model 1, and knowledge and attitudes, which showed significant correlations with infection management behavior, were additionally entered into Models 2 and 3, respectively. Nominal measures such as age, grade level, sex, living with family, experience of clinical practice, and experience of PPE-related training were converted into dummy variables. Multicollinearity and residual diagnostics were conducted for basic hypothesis testing using a linear regression analysis. As a result, with the Durbin–Watson statistic standing at 1.833, there was no autocorrelation, and with the variance inflation factor (VIF) ranging from 1.05 to 4.75 (thus, not exceeding 10), there was no collinearity problem. To verify the assumption of normality and equality of variance of the error terms through residual analysis, the normal PP curve and residual scatterplot were examined. With the independent and dependent variables found to have normal distributions, the regression analysis results were confirmed to be valid.

Analysis of the independent variables for ERID-related infection management behavior identified ERID-related attitudes (β = 0.554, *p*<0.001) and knowledge (β = 0.282, *p*<0.001) as the most important predictors. With the final explanatory power of this model estimated at 40.2%, the regression model was statistically significant (F = 27.935, *p*<0.001) (Table 6).

**Table 6:** Factors influencing ERID-related infection management behavior (N = 481).

Variables	Model 1			Model 2			Model 3		
	β	t	*p*	Β	t	*p*	β	t	*p*
**Age (years)**									
<20 (ref.)									
20–29	0.267	2.768	**0.006**	0.261	2.81	**0.005**	0.167	2.162	**0.031**
30–39	0.191	2.494	**0.013**	0.182	2.473	**0.014**	0.084	1.368	0.172
>40	0.177	2.318	**0.021**	0.167	2.273	**0.023**	0.108	1.764	0.078
**Grade**									
1st (ref.)									
2nd	0.132	2.151	**0.032**	0.091	1.528	**0.127**	0.11	1.528	0.127
3rd	0.096	1.097	0.273	0.095	1.136	0.257	0.132	1.136	0.257
4th	0.057	0.649	0.517	0.069	0.809	0.419	0.13	0.809	0.419
**Sex**									
Male (ref.)									
Female	0.084	1.847	0.065	0.044	0.997	0.319	-0.011	-0.289	0.773
**Living with family**									
No (ref.)									
Yes	0.109	2.396	**0.017**	0.062	1.383	0.167	0.043	1.176	0.24
**Clinical practice**									
No (ref.)									
Yes	0.034	0.38	0.704	-0.005	-0.054	0.957	-0.045	-0.63	0.529
**PPE** ^a^ **-related training**									
No (ref.)									
Yes	0.071	1.384	0.167	0.081	1.637	0.102	-0.004	-0.1	0.921
**Knowledge**				0.282	6.336	**<0.001**	0.138	3.61	**<0.001**
**Attitude**							0.554	14.612	**<0.001**
	**R**^**2**^ **= 0.079,**			**R**^**2**^ **= 0.152,**			**R**^**2**^ **= 0.417,**		
	**Adjusted R**^**2**^ **= 0.059, F = 4.027, *p*<0.001**			**Adjusted R**^**2**^ **= 0.132, F = 7.615, *p*<0.001**			**Adjusted R**^**2**^ **= 0.402, F = 27.935, *p*<0.001**		

^a^PPE, personal protective equipment

## Discussion

This study was conducted to determine nursing students’ levels of ERID-related knowledge, attitudes, and infection management behavior and the factors that affect ERID-related infection management behavior.

Nursing students’ ERID-related knowledge level score was approximately 18 out of 20, that is, 90 points on a scale of 100. This was considered to be relatively high, as in the results of studies that measured knowledge, attitudes, and behaviors related to COVID-19 among nursing students in Italy [14] and nurses in Indonesia [15]. However, in other studies based in Korea, lower knowledge scores, compared to the scores derived in the current study, have been reported: 76.4 points obtained in a study [16] of nursing students’ knowledge level regarding MERS, and 85 points obtained in a study [11] that measured healthcare students’ COVID-19 related knowledge. In a previous study that measured MERS-related knowledge [16], clinical nurses caring for patients may not have taken much interest in new ERID-related information, given the situation in South Korea, where the outbreak of MERS had an infection pattern limited to nosocomial infection, unlike the community infection of the currently prevalent COVID-19 pandemic. The lower score in the study that measured healthcare students’ COVID-19 related knowledge [11] may be explained by the fact that the survey was conducted in an early phase of the COVID-19 outbreak. In contrast, the present study began in 2022, when global vigilance against ERID had peaked. In the case of Korea, the government and media provided disease-related and epidemiological information, including patient trajectories through social media and other forms of mass media, which contributed to the high knowledge scores obtained by the nursing students who participated in this study. In particular, the correct answer rate for “Hand washing with water and soap for at least 30 s can help prevent the spread of ERIDs” was the highest at 99.6%. This may be because hand washing and distancing are being emphasized worldwide due to the COVID-19 pandemic. However, a notable aspect not only in the results of this study but also those of previous studies [14, 15] is that even though the overall knowledge score for ERIDs was high, the error rate for specialized and detailed knowledge items was high. In particular, low correct answer rates were shown for the items “All ERIDs can be treated prophylactically with vaccines” (false; 58.2%) and “The criteria for lifting a quarantine for confirmed COVID-19 cases vary depending on the patient’s symptoms, whereby the absence of clinical symptoms is usually judged as no risk of contagion” (false; 49.3%). This may be due to the lack of proper, professional, and elaborate knowledge and education on ERIDs among nursing students. In fact, 74.4% of the participating nursing students reported that they had received ERID-related education, 425 out of 481 (87.7%) students answered that they were receiving continuously updated ERID-related information through the Internet and TV, and only 30 (6.2%) answered that they were receiving professional information from their instructors. This highlights the need to update and supplement ERID-related information periodically to provide elaborate and professional education on the causes, transmission routes, symptoms, treatment modalities, and quarantine measures.

Nursing students’ attitudes toward ERIDs had a mean score of 4.01 on a 5-point scale, which is similar to the 3.75 points scored by nurses who participated in Choi and Lee’s study [10] regarding attitudes toward MERS. Nursing students, as the future nursing workforce, obtained high attitude scores for items on PPE, which should be prioritized for the direct nursing care of infectious disease patients. They answered that they would always work with PPE in situations such as ERID outbreaks because PPE can protect the wearer from infection. Nevertheless, nurses have also reported experiencing extreme physical and mental stress while wearing protective gear during the COVID-19 pandemic [7, 17, 18]. This is attributed to the fact that the rapidly increasing number of patients due to COVID-19 led to a shortage of PPE such as N95 masks and protective clothing necessary for caring for patients with infectious diseases and that nurses had to provide nursing care with unfamiliar protective clothing in an unprepared state without prior training, which led them to experience physical and mental stress [7, 17, 18]. Therefore, care should be taken to provide nurses with practical training directly applicable to clinical settings, such as donning and removing protective clothing, properly wearing a mask with tightness testing, and taking a test sample from a suspected patient, in addition to ERID-related knowledge education.

Regarding ERID-related infection management behavior, the participating nursing students achieved a mean score of 4.67 on a 5-point scale, exceeding 4 points in all items, which was evaluated to be excellent. This was consistent with the result reported by a nursing student in a previous study on good practices toward virus transmission prevention [14–16]. These results reflect the current situation that even without nursing experience, nursing students have come to realize the importance of infection management, based on their experiences of an ERID pandemic with the COVID-19 outbreak since 2019, and that they are highly willing to make efforts to ensure effective infection management in case of recurrent ERIDs in the future.

For all items on ERID-related knowledge, attitudes, and infection management behavior according to general characteristics, female students generally outscored male students, presumably due to a higher risk sensitivity to infectious diseases in women than in men [19], which was also reflected in the higher scores for ERID-related knowledge, attitudes, and infection management behavior obtained by female students in the nursing department, where women far outnumber men. In addition, significant differences were observed in knowledge, attitudes, and infection management behavior scores according to age, grade level, living with family status, experience of clinical practice, and experience of PPE-related training. In the curriculum of South Korean nursing schools, freshmen are primarily exposed to liberal arts subjects, and major subjects are taught from the second year onward, including infection control education, which explains the score differences in ERID-related knowledge and infection management behavior between freshmen and other grade levels. As for the higher score obtained by students living with family, it can be assumed that anxiety about the risk of infection transmission among family members drives a greater interest among nursing students living in a family regarding ERID-related knowledge and attitudes than those living alone, which is in line with the results of a previous study [20] that demonstrated that people living with family respond more sensitively to ERIDs because of increased anxiety about infection transmission among family members. In the items for ERID-related infection management behavior, students with experience of clinical practice and PPE-related training scored higher, which may be associated with the clinical practice provided to junior and seniors, mostly including PPE-related training.

The results of this study on the correlations between ERID-related knowledge, attitudes, and infection management behavior revealed that the higher the knowledge about ERIDs, the more positive the attitudes toward ERIDs and the higher the correlation with ERID-related infection management behavior. According to the rational behavior theory of Ajzen and Fishbein [21], an individual’s attitude changes with changes in knowledge, and positively changed attitudes influence behavior, leading to positively changed behavior. Therefore, to improve nursing students’ ERID-related infection management behavior, it is necessary to enhance their knowledge and foster positive attitudes. In addition, an analysis of the factors influencing ERID-related infection management behavior revealed attitudes toward ERIDs as the most important variable, followed by knowledge of ERIDs. Therefore, an infection control education program that can foster positive attitudes and provide accurate knowledge regarding ERIDs can be an effective way to prevent and control infectious diseases, ultimately strengthening nursing students’ ERID-related infection management behavior [22–24].

## Limitations

The limitations of this study are as follows. First, the results of this study have limited generalizability because the data were collected from nursing students attending a nursing school in South Korea. Therefore, future studies should consider data collection methods other than convenience sampling when recruiting participants, which will be helpful to establish the generalizability of the results. Second, since the evaluation of ERID-related knowledge, attitudes, and infection management behavior was not performed objectively but through a self-report questionnaire, the possibility of overrating cannot be ruled out. Third, since most of the participants in this study were female and living with family, there is the possibility of sex/gender and sociodemographic biases. Finally, since data were collected from Korean students, the results of the study on nursing students’ other conditions may differ due to cultural influences. Despite these limitations, this study is significant in that it provides basic data for developing an ERID infection control education program for nursing students and also provides an opportunity to review and re-establish the adequacy of infection control education currently provided to nursing students.

## Conclusions

This study was conducted to determine nursing students’ ERID-related knowledge, attitudes, and infection management behavior and to determine the factors influencing infection management behavior. The results of this study demonstrated that nursing students have high levels of ERID-related knowledge and attitudes. ERID-related infection management behavior was found to be most influenced by attitudes and knowledge. Therefore, to improve ERID-related infection management behavior in nursing students in the future nursing workforce, their ERID-related knowledge and attitudes will have to be enhanced by repeatedly providing them with accurate and professional information about ERIDs. Thus, in future research, intensive efforts should be invested in developing a systematic educational program that can help nursing students better perform infection management when caring for patients with infectious diseases in their careers as clinical nurses.
